# Successful Surgical Management of Congenital Prepubic Sinus

**DOI:** 10.4274/balkanmedj.2016.0538

**Published:** 2017-01-05

**Authors:** Levent Duman, Çağrı Savaş, Coşkun Özbiçer, Sema Bircan

**Affiliations:** 1 Department of Pediatric Surgery, Süleyman Demirel University Medical School, Isparta, Turkey; 2 Department of Pathology, Süleyman Demirel University Medical School, Isparta, Turkey

Congenital prepubic sinus (CPS) is a rare anomaly characterized by a sinus tract extending from the prepubic area to the anterior bladder wall. Herein, we present a further case of CPS.

A 20-month-old boy was admitted to our hospital with a 3-month history of recurrent mucopurulent discharge from a pinpoint opening located over the prepubic area (Figure 1a). He had no voiding problems. Physical and laboratory findings were normal. Soft-tissue ultrasound (USG) revealed a 26x9 mm cystic lesion containing loculated fluid collection in the prepubic soft tissue. Magnetic resonance imaging (MRI) (Magnetom Avanto 1.5T; Siemens, Erlangen, Germany) showed a sinus tract with no communication with the urinary system (Figure 1b). A retrograde urethrogram also showed no communication between the fistula and the urinary tract (Figure 1c). During the operation that ensued, a 3 cm-long tract running closely to the pubic symphysis and extending toward the bladder was totally resected (Figure 1d). Histopathologic examination showed that the sinus was lined by only a stratified squamous epithelium and surrounded by bundles of smooth muscles (Figure 1e).

As the number of reported cases of CPS has steadily increased, its embryology is still unclear. The condition was first described as a variant of dorsal duplication of the urethra (1). The presence of a squamous epithelium in or near the skin and a transitional epithelium in the blind deeper part of the sinus with surrounding smooth muscle reinforces this theory (2). But the presence of only a squamous epithelium in our case does not support this theory. The other two theories are that it is a mild anomaly of a midline abdominal wall closure defect, or that it is a congenital fistula of the primitive urogenital sinus (3,4). A recent popular theory states that CPS may be caused by a residual cloacal membrane and umbilicophallic groove (5).

CPS is usually asymptomatic in neonates, and a diagnosis is often made late after extrusion of pus from the sinus as in our patient. The diagnosis is confirmed by detection of the sinus tract extending from the sinus opening toward the anterior bladder wall by USG, fistulography or MRI. Retrograde urethrography and cystography are other diagnostic methods that are used to verify any communication between the sinus and urinary system. On the other hand, the present case and previously reported cases demonstrated that the sinus never communicates with the urinary system. In this respect, we can speculate that retrograde urethrography and cystography are non-essential diagnostic procedures for CPS.

In conclusion, CPS is a rare anomaly with variable presentation. The presentation is often late with minor complications. Complete excision of the fistula is curative, and successful surgical management of CPS requires accurate preoperative assessment of the course of the fistula track.

## Figures and Tables

**Figure 1:**
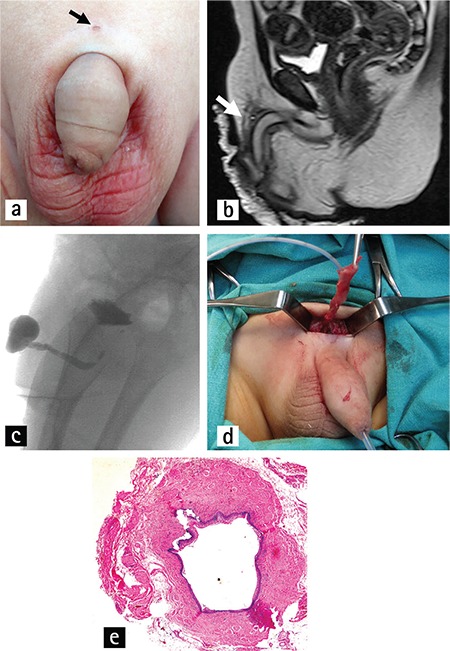
a-e. Sinus orifice (arrow) over the prepubic area (a). MRI revealed a prepubic sinus track (arrow) (b). No communication was observed between the fistula and urinary tract using retrograde cystography (c). Perioperative photo showing a sinus tract extending toward the bladder (d). Histopathologic examination showed that the sinus was lined by only a stratified squamous epithelium and surrounded by bundles of smooth muscles (H&E staining, x40) (e).
